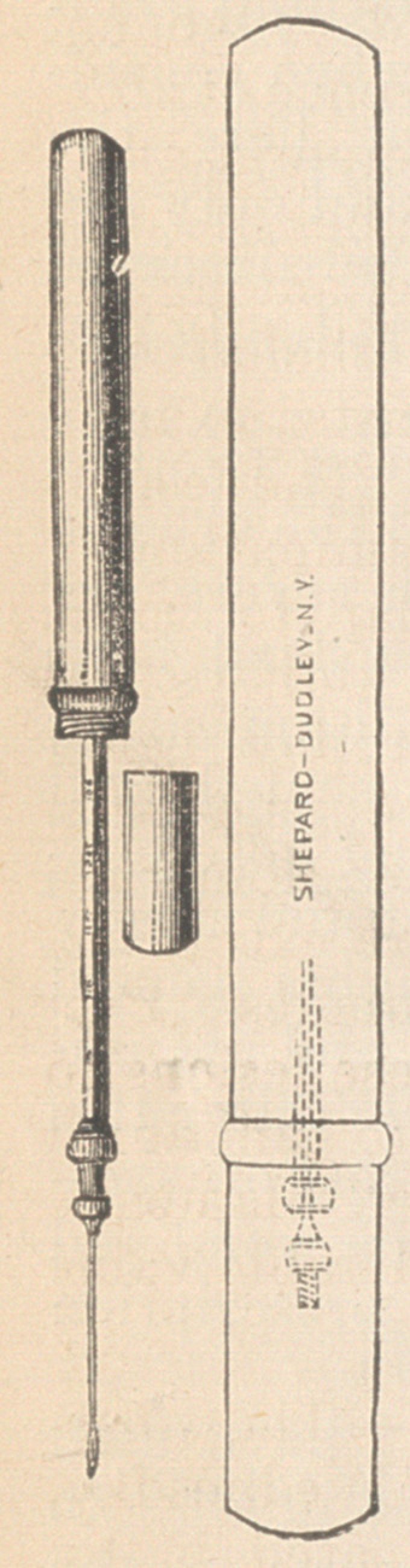# Gleanings from Our Exchanges

**Published:** 1873-11-15

**Authors:** 


Ergot in Nervous Diseases.—Daniel H. Kitchen, M.D., Asst. Phys. to the N. Y. State Lunatic Asylum (*Am. Jour. Insanity*, July, 1873), after repeated investigations, finds that ergotine and Squibb's fluid extract of ergot are beneficial in nervous diseases. In the following forms of headache he has used ergotine with much benefit and comfort to the patient: 1. Headache depending on plethora or fulness of blood. 2. Headache from anæmia. 3. Headaches depending on changes in brain-substance and the membranes. 4. Epileptic headaches. 5. Migraine. 6. Headache depending on disordered menstruation. The author presents ten cases of epilepsy and insanity in which marked and beneficial effect was noticed following the use of Squibb's fluid extract of ergot and ergotine, either alone or in combination with sulphate of quinine and other remedies.

Ergotine may act in two ways: first, directly on muscular fibre, in the same way as any other stimulant; second, through the nervous system, principally the ganglionic. The immediate effect of ergotine on the blood-vessels is marked and rapid; the pulse is increased in force and volume; the slow and wavering pulse becomes full and strong. The power of ergotine is manifest, from its value as a hæmostatic in reducing the size of blood-vessels.

### Gleanings from Our Exchanges.

THE TREATMENT OF ASTHMA.—The following extract is from a lecture by Dr. C. T. Williams, reported in the *Lancet:*

1. *Stimulant Anti-spasmodics.*—A certain number of asthmatics gain relief by sulphuric ether, by strong coffee, and even alcohol. One of the most recently introduced of this class is the nitrite of amyl, which, by acting as a violent cardiac stimulant, sometimes relieves the spasm.

2. *Sedative Anti-spasmodics.*—This is an almost endless class; and if the sufferer perseveres he generally gets relief from one or more members of the group, which includes datura stramonium, tatula, belladonna, hyoscyamus, tobacco, lobelia, Indian hemp, and others. With regard to stramonium, belladonna, and henbane, their use was dictated, as is well known, by the experiments of Dr. C. J. B. Williams, who found that in animals poisoned by these drugs the bronchial tubes were dilated and incapable of being excited by any stimulus, and presented a marked contrast to the results of opium-poisoning, where the bronchial tubes were found contracted after death. Both stramonium and belladonna seem to act most beneficially in the slighter cases; but where they cause dryness of the throat, or even delirium, they seem to have little effect on the bronchial muscle. I found far more satisfactory results follow the use of the extract of stramonium made from the seeds, in quarter grain to half grain doses, than in smoking the cigarettes of the leaves, which, however, have a high repute. Lobelia is a valuable agent when used in sufficiently large doses; but to do much good it must be administered, not in doses of from twenty to thirty minims, as is often done, but a drachm at a time, and repeated every three or four hours until some effect is produced. Tobacco, when smoked, has a sedative influence, but when taken into the stomach is a very unsafe remedy, it being necessary to push it to the extent of vomiting before relief comes; and ofttimes alarming symptoms of failure of the circulation follow. Indian hemp has been used, and with a certain amount of success; but it occasionally gives rise to curious head-symptoms, and therefore requires careful watching.

Inhalations of various kinds, and smoking the different kinds of cigarettes, often do good, if the spasm be not very severe; but when this is the case we must expect them to fail, as they generally do, from the great difficulty of introducing them into the system. It is then that hypodermic injections of morphia come in useful, and by inducing slumber, relax the spasm. The dose should be from one-sixth to one-fourth of a grain, repeated from time to time, and their use is at once contra-indicated by any blueness of the face, or signs of obstructed circulation.

In very obstinate cases you will find that sometimes all drugs fail, chiefly on account of the difficulty of introducing them into the system; and the fit gradually wears itself out, generally through the carbonic acid accumulating in the lungs and inducing anæsthesia of the mucous membrane, and thus relaxing the spasm.

I can testify highly to the benefits of chloroform inhalation in the worst cases, in many of which it has acted like a charm, and sent the sufferer into a calm slumber, from which he has awoke free from dyspnœa. In others the relief is temporary, and the spasm returns as severely as ever. The objections to the use of chloroform seem to be twofold. First, it sometimes, in small quantities, i.e., less than one drachm, causes intermittence of the pulse; second, it cannot with safety be entrusted to the patients themselves.

For these reasons I determined to follow Professor Biermer's example, and try the nearest and safest approach to chloroform, viz.: chloral; and I selected for the purpose several cases where the fits were of long continuance, and marked by only short intermissions. The chloral was given in doses of from fifteen to twenty grains, in an ounce of peppermint water, every three or four hours. The effect, in almost every instance, was that the patients fell fast asleep after the first dose, and slept in a recumbent posture for a few hours, which they had not been able to do for days and weeks. On awaking there was a tendency of the spasm to return, which was generally obviated by two or three repetitions of the large doses. The breathing gradually became quite free, except when considerable emphysema existed: and here the asthmatic spasm was removed, and only the habitual dyspnœa remained. The doses of chloral were then reduced, and gradually discontinued.

Having succeeded with the most troublesome form of asthma, I next tried the drug in cases where the attacks, though severe, are far from periodic, appearing at a fixed hour every night, and completely disappearing every day. Here the chloral seemed still more successful, as a large evening dose generally afforded a good night's rest and steady perseverance with the night draughts seemed to break the habit of the attacks, and eventually to get rid of them altogether. After this I have tried chloral in most forms of spasmodic asthma, and it is not to be wondered at that it has become a favorite prescription in my wards.

In the twenty cases in which I have used it, speedy relief has been obtained in all. In two this has only been temporary; in the other eighteen permanent. In three cases where the scruple doses have been repeated several times, the patient complained of queer sensations in the head; and in one of these, a very obstinate case, where the chloral was continued for days, the patient complained of feeling muddled. Vomiting came on, which disappeared on the appearance of purpuric eruption on the legs. The drug was of course discontinued, and in a few hours the wheezing, which had ceased, returned. This was the only instance of bad effects following.—*Philadelphia Medical Reporter.*

A New Hypodermic Syringe, by Ephriam Cutter, M.D, Woburn, Mass. — Although hypodermic medication has not superseded the gastric, still, for its efficiency, promptness, and energy, it ranks as one of the great advances in modern medicine. It is apprehended that, with the mass of the profession, it is employed mainly when medication by the stomach fails, and in urgent cases only. Be this as it may, in my own experience I

have found that whenever I desired and most needed my hypodermic syringe, it was generally left at home, because the bulk of the box containing it (small as it is) was still found to be cumbersome. This being the case, the writer has sought to contrive a form of syringe which should be so compact as to be carried in the pocket-case of medicines, and occupy the space usually allotted to a phial. The accompanying figures represent in full size the result of this endeavor.

One cut shows the outline of the full-sized instrument, closed. Dotted lines represent the internal arrangement. The other figure illustrates the same with syringe filled, piston drawn out and needle attached. The third figure is a representation of the cap that covers the needle to protect it from harm. The points in which this syringe differs from the ordinary ones are:

1. Closure of the distal end of the cylinder. This makes the syringe a *cul-de-sac*. The circulation of the air is quite different from that in the cylinder of the ordinary syringe. Instead of the air drying the leather of the piston on both sides, it only dries it on one side—thus reducing the chances of drying fifty per cent. Practically this syringe has kept in good order for months continually, when the ordinary syringe would be loose and dry. The drop of moisture confined in the *cul-de-sac* behind the piston has no connection with the air except through the piston; hence it evaporates slowly. Another reason for closing the distal end of the syringe was to keep out dirt and foreign substances.

2. Making the piston and its head hollow throughout, and having a male screw and milled head on the proximal end. By these means a communication is effected with the cavity of the syringe.

3. Having the needle attached to the proximal end of the piston. This latter arrangement is *the* feature which is novel and which constitutes its peculiarity. The instrument is thus complete and ready for use. It is only necessary to draw out the piston with the needle inserted into the medicament. The fluid readily fills the barrel by running through the needle and piston, and the syringe is charged for use.

The method of introduction employed by Dr. F. F. Brown of Reading, Mass., is good. He pinches up a fold of the skin, on the arm or leg or elsewhere, between the left thumb and fore-finger, then holding the needle point in the right hand vertically, enters the point into the integument pinched in the angle included between the finger and thumb-tips. The point is pushed in until the milled head of the piston touches the finger, then the cylinder of the syringe is pushed down and the injection is introduced. The instrument is taken out by withdrawing the piston. When this is done, then, and only then, is the fold of skin relaxed from its hold.

After use, a few movements of the piston to and fro will clear the instrument of the superfluous fluid. It is a good plan to leave some water in and keep the piston head moist (Indeed, this is a good practice for any syringe. Since adopting the habit of keeping syringes half or wholly filled with water when not in use, I have had little trouble with them).

The needle should then be unscrewed and put point foremost into the cavity of the piston. The piston is pushed home, and the cap screwed on. The instrument is now ready to be laid aside in the medicine pocket-book, or in the vest pocket. It will be seen that this instrument is much simpler than others; and yet it is as effective, and much more portable.

The writer is greatly indebted to the firm of Shepard & Dudley, 150 William street, New York city—the makers of the instrument—for suggesting the new alumina alloy as a material for the needle. Steel gold-plated needles rust when kept in this instrument, but this difficulty is overcome by the new alloy, which will not rust or tarnish.

The firm mentioned above supply two forms of this instrument: one of German silver, nickel-plated, with alumina alloy needle, price two dollars; and the other entirely of the alloy, price three dollars.

In closing, the writer would say that he prefers extemporaneous solution of morphous salts for subcutaneous injections. The bottom of a glass goblet or tumbler, turned upside down, furnishes a clean and handy cavity in which to deposit the salt. The syringe may be filled with water and its contents discharged on the salt. The circulating motion thus given aids in dissolving the salt, and then it can be taken up into the syringe for use.

If those who use this syringe experience the same gratification as the writer, it will be an ample reward for his pains in this matter. —*Boston Journal of Chemistry*.

Compression of the Brain.—S. W. Gross, M.D. (*Am. Jour. Med. Sciences*, July, 1873), remarks that the treatment of compression from depressed bone is still a matter of much dispute. That a man may recover from a very considerable and very unequal displacement of the tables of the skull, is attested by numerous examples; but that such cases survive in greater proportion than those in which the pressure has been relieved by the resources of art, is another and highly important question which has not been sufficiently examined by writers on army surgery. Of 224 depressed gunshot fractures of the skull, and in 90 operative measures, of these 45 or 50 per cent. died. Of 134 instances, on the other hand, in which the treatment was purely conservative and antiphlogistic, and in 43 of which the signs of compression were very doubtful, 61 recovered, and 73, or 54.47 per cent. died; or if the doubtful cases, which resulted in 10 deaths, be excluded, 91 cases of compression from depressed fractures, treated expectantly, afford 63 deaths, or a mortality of 69.23 per cent., a result in favor of operation by 19 per cent.

These statistics show, that when symptoms result from depressed bone, the chances of saving life are on the side of surgical interference. The trephine was applied in 49 of the above 90 operations, with 26 deaths, the mortality being 53.06 per cent.; fragments of the shattered bone were removed by the forceps in 28 instances, with a fatality of 14, or 50 per cent.; the elevator was resorted to in 10, of which 3, or 30 per cent. were mortal; while in 3 instances Hey's saw was followed by death in 2, or 66.66 per cent. An examination of these facts does not disclose that the operation of trephining is more dangerous than other operative procedures. More patients recover after than without surgical interference, and many lives might have been saved by a timely resort to it.—*Med. Record.*

Chloroformization During Sleep.—Dr. W. M. Whitmarsh states (*Lancet*): "Having occasion to perform circumcision on a very nervous child, aged six years, and the evening being selected by the parents for the operation, I found on my arrival the little patient fast aleep. Not wishing to lose so good an opportunity, I, with my friend Mr. Gandy, thought it advisable to administer chloroform at once. This was done by pouring ten drops on a piece of lint, and repeating it until one drachm had been given, when the patient was thoroughly under its influence. The operation was then performed, and the patient dressed, not waking till half an hour after. The pulse did not appear to differ from that ordinarly observed during the administration of chloroform. It would be interesting to know if this mode of giving chloroform has been noticed by the profession, and whether in nervous patients and young children it would not be preferable to the shock to the system occasioned by fright and fear of suffocation."—*The Clinic.*

Albuminuria as a Symptom of Meningitis.—Professor Rosenstein has noticed the coincidence of albuminuria with meningitis. The analysis of their urine demonstrates in nearly all patients, whether old or young, attacked with meningitis, the presence of albumen in the early days of the cerebral affection; and in a considerable number of these cases is found at the same time, among the products of the renal secretion, epithelial cells, blood-globules, and fibrinous casts, exactly the same as in Bright's disease. The lesions discovered by post-mortem examination of the kidneys are likewise similar to those of albuminous nephritis. The kidneys are increased in size—the hypertrophy being chiefly in the cortical substance; the renal tissue is markedly injected; the glomerulæ gorged with blood, and there are extravasations of blood into the urinary tubules. Professor Rosenstein attributes these lesions to troubles in the circulation, due to functional alteration of the vaso-motor nerve filaments. This fact is likely to be of considerable value in diagnosis.—*Lyon Med.*

The Funeral of Nelaton.—The obsequies of Nelaton were held on Wednesday, as announced, and were participated in by throngs of people of all ranks of society. The cortege was set in motion at noon precisely, headed by M. Charles Nelaton, son of the deceased, who will attempt to follow in the footsteps of his father to fields of distinction in surgery. The pall-bearers were M. Bouillaud, representing the Institute; M. Bouchardat, for the Medical Faculty; M. Depaul, President, and M. Beclard, perpetual Secretary of the Academy of Medicine. The Society of Surgery, the Association of Physicians of the Seine, were represented by a great number of members. The religious ceremony was held at the Church of St. Pierre de Chaillot. Military honors were rendered to this grand officer of the Legion of Honor by a batallion of the line. According to Nelaton's expressed desire, no discourse was pronounced at the funeral ceremony over his grave.—*Gazette des Hopitaux*, Sept. 25, 1873.

American Medical Graduates.—The various American Medical Colleges have graduated this year about 1200 students.

PNEUMATIC ASPIRATION IN HYDRARTHROSIS OF THE KNEE-JOINT.—Dr. Rasmussen, of Copenhagen, describes seven cases in which he practiced aspiration in eight knee-joints. In none of these cases did the slightest trace of inflammatory reaction follow the operation. Even in the cases where there had been severe pains and considerable tenderness of the joint, it was so free from both for several days after the operation that treatment by *massage* could be employed for the removal of the remainder of the effusion. He advises the operation in both the chronic and acute forms of this affection.

The following is Dr. Rasmussen's mode of operating: Broad strips of adhesive plaster, clipped at the ends, are applied above and below the joint, and are then gradually tightened according as the evacuation of the fluid by suction, which takes place very slowly, proceeds. By the continued application of adhesive plaster, the fluid is forced towards the canula.

This should, as a rule, have a diameter of from .08 to .06 of an inch, so that the viscid portion of the fluid can pass through it. At first, Dr. Rasmussen made the puncture through the extensor muscles, in the highest pouch of the capsule, for fear that the fluid would continue to ooze through the comparatively large opening if the latter were to be made at a lower level. This subsequently, however, was found to be both unnecessary and inexpedient, for by this plan the fluid could with difficulty be all drawn off, and the puncture is now made in an upward direction at the external edge of the patella. After the removal of all the fluid, the opening is closed with charpie and collodion, and the last strip of plaster is applied in the center, and thus the joint is compressed and placed at rest. To secure rest more completely, the leg is bandaged and an ice-bladder is applied to the knee, although the latter is thought to be, perhaps, superfluous.

Occasionally slight œdema of the foot and ankle follow the operation; but clipping of the plasters, and so loosening them, causes it to disappear. The bandage may be removed at the end of three or four days, and the fluid will commonly have disappeared. When it has recurred it is in less amount, and the joint is quite free from pain.

The utmost care is essential in disinfecting the trocar, and in operating in a locality where there is neither pyæmia nor erysipelas.—*Med. Record.*

THORACENTESIS IN SCARLATINAL PLEURISY.—Dr. R. P. Howard, Professer of Medicine in McGill University, publishes in the new number of the *Canada Lancet* four cases, from which he draws the following conclusions:

1. That the pleurisy of scarlatina is usually —not to say invariably—an acute empyema.
2. That in *scarlatinal* pleurisy, when the signs of effusion are marked and do not promptly disappear, it is well to make an exploratory puncture of the chest at a much earlier period than is even now customary in *ordinary* pleurisy following *exposure*.
3. The tolerably prompt and, at the same time, complete recovery of the lung may be expected under these circumstances, chiefly because the inflammation is *acute* and *recent*, and that the vital powers have not been exhausted by a protacted illness, nor the condition of the lung been altered by prolonged compression, as in chronic empyema.
4. That if the disease (the pleuritis) be not of long standing, *i.e.*, if it be recent, the appearance of pus in thoracentesis is not, at least in scarlatinal pleurisy, a very grave indication. The majority of such cases will probably terminate favorably.
5. That the pus in scarlatinal empyena may perforate the lung and be expectorated, and the patient recover promptly and perfectly.
6. That it is not well to wait for such an occurrence, which appears to be unusual, and, as being long delayed, to involve increased danger to life, but rather to make an exploratory puncture early.
7. That if the pyothorax of scarlet fever be recent, simple puncture of the chest repeated once or oftener will usually suffice, without the employment of the drainage tube, which is so valuable and often necessary in chronic pyothorax. I may add that, judging from my experience in other cases, the same observation will apply to other forms of acute pyothorax.

## Figures and Tables

**Figure f1:**